# Empathy in Females With Autism Spectrum Disorder

**DOI:** 10.3389/fpsyt.2019.00428

**Published:** 2019-06-18

**Authors:** Sanna Stroth, Lena Paye, Inge Kamp-Becker, Anne-Kathrin Wermter, Sören Krach, Frieder M. Paulus, Laura Müller-Pinzler

**Affiliations:** ^1^Department of Child and Adolescent Psychiatry, Psychosomatics and Psychotherapy, Faculty of Medicine, Philipps-University Marburg, Marburg, Germany; ^2^Marburg Center for Mind, Brain and Behavior (MCMBB), Philipps-University Marburg, Marburg, Germany; ^3^Social Neuroscience Lab, Department of Psychiatry and Psychotherapy, University of Lübeck, Lübeck, Germany

**Keywords:** female ASD, empathy, social pain, vicarious embarrassment, fMRI

## Abstract

**Objective:** Despite the fact that autism spectrum disorder (ASD) is a common psychiatric diagnosis, knowledge about the special behavioral and neurobiological female phenotype is still scarce. The present study aimed to investigate neural correlates of empathy for physical and social pain and to assess the impact of egocentric perspective taking on social pain empathy in complex social situations in women and girls with ASD.

**Methods:** Nine female individuals with high functioning ASD were compared to nine matched peers without ASD during two functional magnetic resonance imaging (fMRI) experiments, examining empathy for physical and social pain using well-established paradigms. Participants viewed multiple pictorial stimuli depicting a social target in either physically painful or socially unpleasant situations. In the social situations, the participant either shared the social target’s knowledge about the inappropriateness of the situation (observed social target is aware about the embarrassment of the situation; e.g., tripping in public) or not (observed social target is unaware about the embarrassment of the situation; e.g., open zipper).

**Results:** Females with ASD did not rate the physical pain stimuli differently from non-clinical controls. Social pain situations, however, posed a greater challenge to females with ASD: For non-shared knowledge situations, females with ASD rated the social target’s embarrassment as more intense. Thus, compared to non-clinical controls, females with ASD had a stronger egocentric perspective of the situation rather than sharing the social target’s perspective. On the neural systems level, both groups showed activation of areas of the so-called empathy network that was attenuated in females with ASD during empathy for physical and social pain with a particular reduction in insula activation.

**Conclusion:** Females with high functioning ASD are able to share another person’s physical or social pain on the neural systems level. However, hypoactivation of the anterior insula in contrast to individuals without ASD suggests that they are less able to rely on their shared representations of emotions along with difficulties to take over a person’s perspective and to make a clear distinction between their own and someone else’s experience of embarrassment.

## Introduction

Autism spectrum disorder (ASD) is a pervasive neurodevelopmental disorder involving impairments in two core domains: social interaction, including verbal as well as non-verbal communication, and stereotyped, repetitive behaviors and interests (1). Despite the fact that ASD is a common psychiatric diagnosis, with an onset within the first years of life and very early impairments of social attention and reciprocity (2), knowledge about the special behavioral and neurobiological female phenotype of ASD is still scarce. One of the reasons for this imbalance in the literature is the predominance of male ASD cases which is a consistent epidemiological finding. The male-to-female ratio averages at 4–5:1 but increases to about 10:1 in cases of high functioning ASD and decreases to 2:1 in affected individuals with moderate-to-severe intellectual disability (3, 4). Current estimates range from 3:1 to 4:1 across the autism spectrum, but the reason for the consistently observed discrepancy in the sex ratio remains unclear (5). Although neuroanatomical and neurofunctional differences between sexes/genders have been described (6), research on the female peculiarities is still insufficient and results are often inconsistent. One reason for this lack of knowledge about the female ASD phenotype is the striking underrepresentation of females with ASD in neuroimaging research in general with an overall sex ratio of 8:1 (male:female). A recent meta-analysis of 329 articles revealed that only 1 out of 15 functional MRI studies actually included females with ASD (7). Knowledge about relations between biological as well as anatomical factors and the psychosocial impairments in ASD is therefore mainly based on the high functioning male phenotype of ASD. It has been hypothesized that specific aspects of the neuroanatomy underlying ASD may represent phenotypic diversity in brain structure that is specific for males, which could make females more resilient to autism-related social deficits (8). A recent study, however, could not find support for this claim (9), and at present, there might just not be sufficient data available to obtain robust evidence in support of this model. Further, research in mouse models for neurodevelopmental disorders suggests specific impairments in reward-directed learning only in male mice but not in females (10). The relevance of such efforts and generalizability to human social cognition and the ASD phenotype, however, remains unclear. We believe that further research is definitely needed to specify sex/gender differences related to ASD and social cognition. The lack of research on the female phenotype challenges the generalizability of the notion that alterations of processes in the domain of social cognition, such as emotion recognition, empathy, and theory of mind, could explain the observed peculiarities in social interactions in ASD. Here, we take a closer look at the empathic response of girls and young women with ASD to physically (painful) and socially (embarrassing) threatening situations of others. Thereby, we aim to broaden our perspective on the ASD phenotypes and to test specific assumptions about how the complexity of the social situation modulates the empathic response in females with ASD.

One core domain of difficulties in individuals affected by ASD is the domain of social cognition. Processes such as emotion recognition, empathy, and theory of mind (ToM) have been found to be severely disturbed (7). Impairments in these domains impede individuals with ASD to engage in social interaction (11). Many behavioral and neurofunctional studies (with mostly male participants) have demonstrated deviant patterns of empathy-related information processing in individuals with ASD along with diminished activation of brain areas involved in ToM and empathy, namely, inferior frontal gyrus, medial prefrontal cortex, and anterior insula (12–14). It has been argued that individuals with ASD show deficits in sharing another person’s affective state (15, 16). However, such deficits might primarily surface in more complex social situations, in which contextual demands such as knowledge about social norms, expectations of the social environment, and appraisals of the social target need to be dynamically integrated (13, 17, 18).

Sharing another person’s physical pain or empathizing with another person in a socially unpleasant situation (e.g., social exclusion or embarrassment; experiences also referred to as social pain^1^) increases activations in brain regions that are also recruited during the first-hand experience of the same affective state (19, 23, 24). This observation leads to the assumption that we are able to understand others’ emotions based on our own shared affective experiences. For example, when making sense of rather complex social faux pas situations that typically elicit a shared experience of embarrassment with another person, brain areas of the so-called mentalizing network ([medial prefrontal cortex (mPFC) temporal pole, and superior temporal sulcus (STS)]) as well as the anterior insula (AI) and the anterior cingulate cortex (ACC) are recruited (17, 23). A previous study of ours contrasting male participants with ASD to non-clinical controls found the AI, a part of the so-called empathy network typically recruited when sharing another’s physical or social pain (17, 19, 24, 25), to be hypoactivated when experiencing embarrassment on behalf of others (13). However, when sharing another person’s physical pain, male participants with ASD did not reveal diminished neural activation of the empathy network (13). One explanation for these findings is that faux pas tasks are more demanding and require the observer to integrate contextual information, as well as to take into account the social target’s perspective. Specifically, when one’s own knowledge about a situation fundamentally deviates from the social target’s knowledge, adjusting the own view in order to make sense of the other person’s thoughts and feelings is inherently challenging. Since people tend to use their own subjective perspective as an anchor, perspective taking is described as a time consuming and effortful process of adjusting one’s initial view that often results in subjectively biased assumptions and subjectively imbued representations on the neural systems level (26, 27). The more complex a situation gets, the more likely the own perspective remains egocentrically biased. While egocentric anchoring is thought to be a typical process when making sense of another person’s thoughts and feelings, individuals with ASD have been described to show a particular and exaggerated form of egocentrism with clear difficulties to make use of embodied simulation strategies (28) and to overcome their egocentric perspective (29, 30). These difficulties are of specific relevance when people navigate complex social situations and make sense of others’ mental and affective states—that might be distinct from the own experience—in order to adequately engage in social interactions. However, to date there is little knowledge on how females with ASD can adopt another person’s perspective in socially complex situations eliciting empathy for social and physical pain.

With the current work, we therefore aimed to shed light on the peculiarities in empathy-related processes in females with ASD. In line with our previous findings in male adolescents with ASD, we hypothesized that females diagnosed with ASD would also show hypoactivations in brain areas involved in empathic processes in response to complex social situations associated with embarrassment on behalf of others (AI, ACC). Similarly, we did not expect females with ASD to differ from non-clinical controls when empathizing with another person’s physical pain (13). Specifically, when the task requires inferring another person’s affective state, whose knowledge about the situation is different from one’s own knowledge, we expected to see pronounced egocentric biases in females diagnosed with ASD.

## Methods

### Participants

The study was conducted in the specialized outpatient clinic for ASD at the University Hospital for Child and Adolescent Psychiatry, Psychotherapy and Psychosomatic Medicine in Marburg, Germany. The study was approved by the local ethics committee (Az 197/12). Written informed consent was obtained from all participants and parents in case the participants were underage. Female participants with ASD (F-ASD; *N* = 9) were recruited from our outpatient clinic as well as other clinics, and the age span within the F-ASD group ranged from 12.5 to 24.5 years (mean age of 18.7 years). All patients matched the *Diagnostic and Statistical Manual of Mental Disorders, Fourth Edition* (DSM IV) criteria for ASD, had a confirmed International Classification of Diseases, 10th Revision (ICD-10)diagnosis of Asperger syndrome (*n* = 5; F84.5, ICD-10) and/or atypical autism (*n* = 4; F84.1, ICD-10), and had undergone standardized diagnostic procedures with either Module 3 or Module 4 of the Autism Diagnostic Observation Schedule (ADOS) (31). If parental informants were available, the Autism Diagnostic Interview-Revised (ADI-R) (32) was administered. The mean verbal IQ was 112 as confirmed with the Wechsler Intelligence Scale for either adults (WAIS-IV) (33) or children (WISC-IV) (34). The age- and IQ-matched non-clinical control group (F-CG; *N* = 9) had a mean age of 19.9 (range, 13.9–25 years) and a mean verbal IQ of 113 (for additional information, see **Table 1**). Due to technical problems with the response box, behavioral ratings of one control participant had to be excluded from further analyses. All participants had normal or corrected-to-normal vision. F-CG and F-ASD differed significantly (*p* = .015) concerning the self-report evaluation of autistic symptoms as conducted with the Autism Spectrum-Quotient questionnaire (AQ) (35).

**Table 1:** Sample characteristics.

	F-ASD (*N* = 9)		F-CG (*N* = 9)		p	z
	Mean	SD	Mean	SD	(Mann-Whitney *U* test)	
Age	18;7	4;9	19;9	3;6	.667	0.486
Verbal-IQ	112.0	15.0	113.0	9.0	.797	0.265
AQ	22.6	10.1	9.8	2.5	.015	2.368
EQ	111.7	14.8	118.4	6.5	.277	1.157
ADOS-SA	7.7	3.1				
ADOS-RRB	1.0	0.9				
ADOS Comparison Score	4.9	2.1				
ADI-R Com	6.5	2.7				
ADI-R Soc	7.3	4.5				
ADI-R Stereo	2.7	2.0				

AQ, Autism Spectrum Quotient; EQ, Empathy Quotient; ADOS, Autism Diagnostic Observation Schedule; ADOS-SA, ADOS Social Affect Score; ADOS-RRB, ADOS Repetitive and Restricted Behavior Score; ADOS Comparison, ADOS Comparison Score; ADI-R, Autism Diagnostic Interview-Revised; ADI-R Com, ADI-R Communication Score; ADI-R Soc, ADI-R Social Interaction Score; ADI-R Stereo, ADI-R Stereotyped Behavior Score. F-ASD, female autism spectrum disorder group; F-CG, female non-clinical control group; SD, standard deviation; p-values and z-values refer to the Mann-Whitney U test comparing the two groups against each other.

### fMRI Paradigm and Stimuli

**Figure 1:**
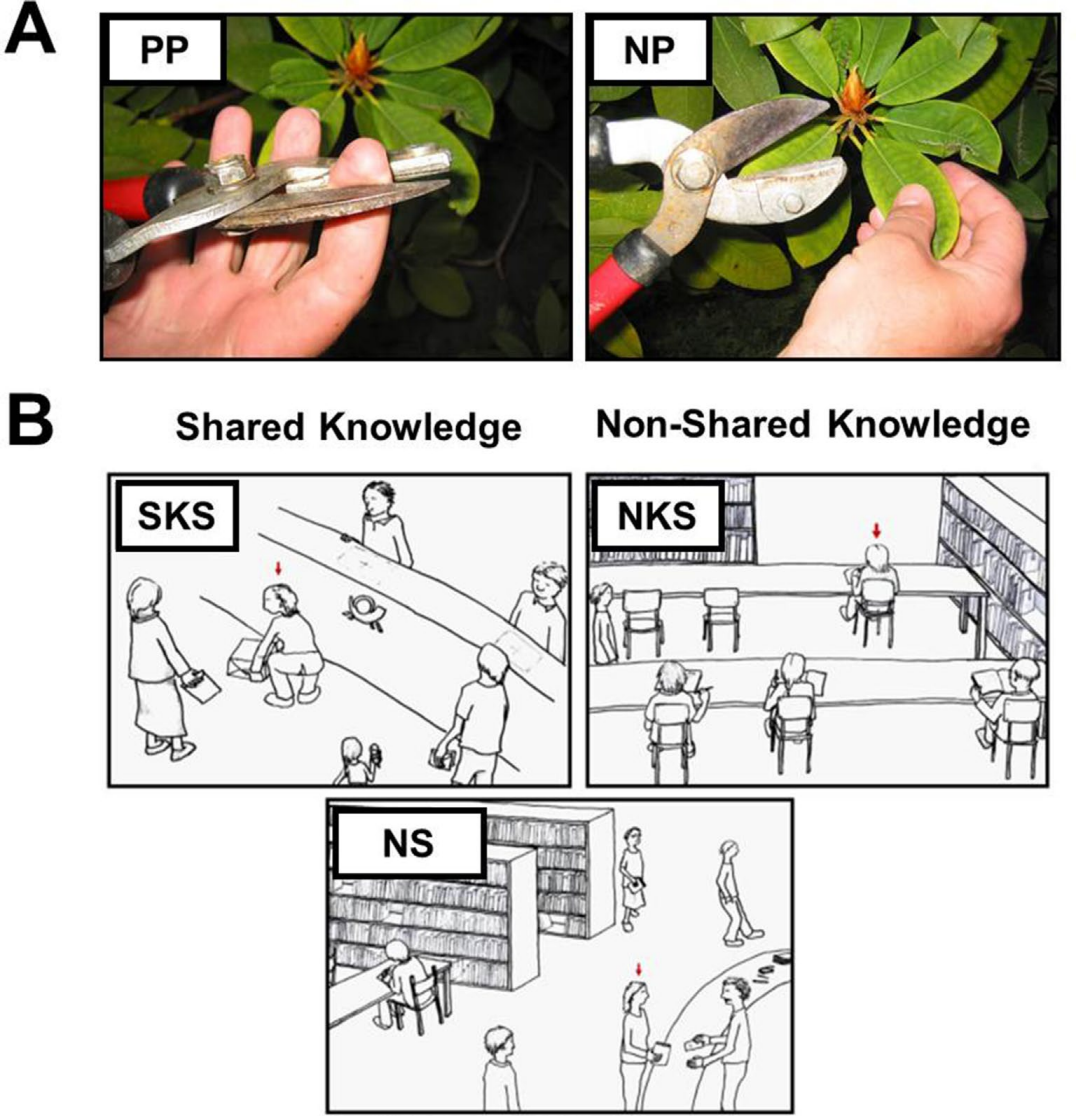
Stimuli used in the functional magnetic resonance imaging (fMRI)experiments. **(A)** Depiction of one example stimulus of the experimental paradigm to induce empathy for physical pain. The physical pain situation (PP) is presented on the left and the neutral, no-pain control situation on the right side [NP; stimuli were taken from Jackson et al. (36)]. **(B)** The situations eliciting empathy for social pain varied concerning the social target’s awareness about the inappropriateness of the situation and thus regarding the shared knowledge and affective experience of the observer and the social target. Ten situations depicted situations with shared knowledge about the inappropriateness of the situation (SKS) eliciting shared affect with the social target, e.g., sharing embarrassment with a person who’s pants rip while she bends down to lift a package, and 10 situations depicted a social target unaware about the faux pas, thus not sharing the same knowledge with the observer [non-shared knowledge situation (NKS)], e.g., a person whose pants unknowingly slipped down while she is sitting on a chair. Ten sketches displayed non-norm-violating control situations [neutral control situations (NS)]. Stimuli were presented together with two sentences describing the situation below the sketches (e.g., You are at a library: you observe a women giving back a book she borrowed).

In two consecutive functional magnetic resonance imaging (fMRI) experiments, we induced empathy for physical pain (EPP) and empathy for social pain (ESP) with stimuli and paradigms that have been previously described and similarly implemented in a study with male participants with a confirmed ASD diagnosis (13, 17). To investigate the neural correlates of EPP, participants viewed 28 color photographs depicting another person’s left or right hand or foot from a first-person perspective in either painful [e.g., foot on a log with an axe landing on top of the big toe; physical pain (PP); 14 stimuli] or non-painful, neutral control situations [e.g., foot on a log with the axe hitting next to it in the wood; no pain (NP); 14 stimuli; see **Figure 1**]. The photographs were chosen from a pool of 56 validated stimuli (13, 36). Stimuli were presented for 4.5 s. Subsequently, a fixation cross on a blank screen was presented for 1.5 s. Participants were then asked to respond within 3 s to the question “How strong is the pain of the observed person in this moment?” and rate the intensity of the depicted person’s pain experience (from 1 “not at all” to 5 “very strong”) on a five-point scale. Following the rating phase, a fixation cross was presented for an average of 6.1 s. Stimuli were presented in a fix pseudo-randomized order with no more than two stimuli from the same condition following each other. In total, the experiment lasted approximately 7 min.

To induce ESP, participants were confronted with 30 validated hand-drawn sketches of social situations displaying a protagonist in either socially undesirable (20 sketches) or neutral public scenarios (10 sketches). Each sketch was explained with a written caption, which introduced the context (e.g., “You are at the theatre”) and the state of the person serving as the social target (e.g., “The actor forgets his lines during the play…”). Ten of the 20 social pain sketches illustrated scenarios in which the social target was aware of the predicament and thus could be perceived to be emotionally engaged and embarrassed in the situation. Here, participants shared the knowledge of the faux pas and the experience of embarrassment with the social target [shared knowledge situation (SKS); e.g., “You are at the grocery store. A person at the cashier realizes that she cannot pay for her purchase”]. The other 10 social pain sketches depicted situations in which the social target was unaware of the social norm violation and therefore would not notice that his or her social integrity was at stake. However, participants would notice the faux pas from an observer’s perspective [non-shared knowledge situation (NKS); e.g., “You are on a train. A passenger walks by with an open pants zipper”]. Thus, the observer would be aware that the social target him- or herself does not experience the unpleasantness of the situation, but could nevertheless experience strong states of embarrassment on behalf of the social target (23). Importantly, these two conditions differently vary the impact of egocentricity in the judgement of the situation; when sharing the knowledge in the SKS condition, there is no need to distinguish the other’s perspective from one’s own, while in the NKS condition, the observer’s knowledge could impact the evaluation of the affective experience in the other, if it is egocentrically biased. This notion originates from classic developmental psychological research claiming that young children are egocentric to the degree that they are only capable of contemplating the world from their egocentric perspective (37–39). It is the NKS condition which requires participants to abstract from their egocentric viewpoint (i.e., an open zipper is always embarrassing) but take the perspective of the target person (i.e., being unaware of the open zipper and thus not feeling embarrassed). Finally, 10 neutral control situations depicted comparable social scenarios lacking the faux pas component [neutral control situations (NS); e.g., “You are at a post office. At the neighboring counter you observe a man who is posting a package”]. The social targets were described as male (i.e., a man) in half of the situations or female (i.e., a woman) in the other half in the sentences below the picture. Participants were instructed to attend to each sketch for 12 s. Stimulus presentation was followed by a blank screen for 1 s and a 3-s rating period during which participants were asked to indicate the social target’s embarrassment on a five-point scale (from 1 “not at all” to 5 “very strong”) in response to the question: “How embarrassing is the situation for the observed person in this moment?”. The stimuli were presented with an inter-trial interval of 8 s in a pseudo-randomized order, which was the same for every participant, with no more than two stimuli from the same condition in a row. The total duration of the experiment was approximately 12 min.

Before entering the MRI, participants received careful instructions about the experimental procedure using two example situations that were not displayed again during the MRI session. In the scanner, a response box was attached to the right leg and the participants’ fingers were placed in the correct position. Stimuli were presented on an LCD screen with Presentation 12.1 software package (Neurobehavioral Systems, Albany, CA, USA).

### Data Acquisition and Analysis

All participants were scanned at 3T (Siemens Trio, Erlangen, Germany) with 36 near-axial slices and a distance factor of 10% providing whole-brain coverage. An echo planar imaging (EPI) sequence was used for acquisition of functional volumes [repetition time (TR) = 2.2s, echo time (TE) = 30 ms, flip angle = 90°, slice thickness = 3 mm, field of view (FoV) = 192, matrix 64 × 64 voxels, voxel size 3 × 3 × 3 mm]. Overall, we obtained 204 volumes for EPP and 340 volumes for ESP. The first seven (EPP) and four (ESP) volumes of each session were discarded from further analyses. To rule out potential anatomical abnormalities, we acquired high-resolution images with a T1-weighed scan comprising the whole brain, employing a magnetization-prepared rapid gradient-echo sequence (3d MP-RAGE) in sagittal plane (176 slices, TR = 1.9 s, TE = 2.52 ms, flip angle = 9°, ascending slices, slice thickness = 1 mm, FoV = 256 mm, 50% gap, matrix 256 × 256 voxels, voxel size 1 × 1 × 1 mm).

Data were analyzed using SPM8 (www.fil.ion.ucl.ac.uk/spm). For each session, brain volumes were corrected for slice timing and head motion and spatially normalized to the standard EPI template of the Montreal Neurological Institute (MNI) using linear and nonlinear transformations of the mean EPI images of each time session. The normalized volumes were resliced with a voxel size of 2 × 2 × 2 mm, smoothed with an 8-mm full-width half-maximum isotropic Gaussian kernel, and high-pass filtered at 1/192 Hz for the EPP and 1/256 for the ESP task.

#### Analysis of Empathy for Physical Pain Data

All behavioral data were analyzed with PASW Statistics for Windows, Version 18.0 (SPSS Inc. Released 2009). Ratings of the social targets’ pain in the EPP paradigm were analyzed using repeated-measures analyses of variance (ANOVAs). For the analysis of pain ratings, a 2 × 2 ANOVA was implemented with Condition (PP, NP) as within-subject factor and Group (F-CG and F-ASD) as between-subject factor.

On the neural systems level a fixed-effects general linear model (GLM) was calculated at the within-subject level for EPP in order to test for activation differences. The model for EPP included three regressors modeling the hemodynamic responses to the PP, the NP condition, and the rating period with the aforementioned stimulus durations. The PP events were additionally weighted with the corresponding rating response to align our analyses to previous experiments (13, 17). Six regressors modeling head movement parameters were introduced to account for noise. At the group level a flexible factorial design was implemented with condition as a repeated-measures factor (included β-maps for the PP and NP condition) and group as a between-subject factor (F-CG and F-ASD group). Previous results specifically stress areas of the so-called empathy network, the AI and the ACC, as key regions in processing EPP and ESP. Functional regions of interest (ROIs) were defined by deriving activation maps from previous studies using the same stimuli in samples of non-clinical control subjects. The physical pain ROIs were defined according to a previous study of our group assessing EPP contrasting PP-NP (13): ACC, left AI, right AI, and the somatosensory cortex. All ROI analyses were conducted using the small-volume correction as implemented in SPM8, applying voxel based family-wise-error (FWE) correction. Additionally, whole-brain analyses were conducted to assess activations outside of our ROIs, FWE corrected for the whole brain.

#### Analysis of Empathy for Social Pain Data

On the behavioral level, ratings of the social targets’ embarrassment in the ESP paradigms were analyzed using a repeated 2 × 3 ANOVA the Condition (SKS, NKS, NS) as within-subject factor and Group (F-CG, F-ASD) as between-subject factor.

On the neural systems level, as for the EPP task, a fixed-effects GLM was calculated at the within-subject level. For the ESP, the first-level model included one regressor for the SKS, one for the NKS, one for the NS, and another regressor for the rating period. The trials in the SKS and NKS condition were additionally weighted with the corresponding rating response similar to previous approaches (see above), and six additional regressors modeling head movement parameters were included to account for noise. Beta-maps for the SKS, NKS, and NS condition for the F-CG and F-ASD group were analyzed at the group level using a flexible factorial design with condition as a repeated-measures factor and group as a between-subject factor. Functional ROIs for ESP were also defined according to activation maps of a previous study of our group assessing embarrassment on behalf of others with the respective contrast social pain vs. NS in a sample of subjects without ASD (23): ACC, left AI, and thalamus. Corrections for multiple comparisons and whole-brain analyses were conducted as described above for the EPP task. All anatomical coordinates are reported in MNI standard space.

## Results

### Empathy for Physical Pain

On the behavioral level, participants in both groups rated that the social target experienced more pain in the PP situations as compared the NP situations as indicated by the main effect of Condition (*F*_(1,16)_ = 513.08, *p* < .001). F-CG and F-ASD both rated the protagonist’s physical pain as more intense for PP compared to NP (see **Table 2** for comparisons), and the general intensity level of pain ratings as well as the responsiveness to PP vs. NP did not differ between groups (main effect of Group: *F*_(1,16)_ = 0.18, *p* = .679; Group × Condition interaction: *F*_(1,16)_ = 1.43, *p* = .249).

**Table 2:** Behavioral results.

Contrast	Overall (*N* = 17)		F-CG (*N* = 8)		F-ASD (*N* = 9)	
	*F*	*p*	*t(7)*	*p*	*t(8)*	*p*
**Empathy for physical pain**						
PP vs. NP	513.08	<.001	23.07	<.001	12.53	<.001
**Empathy for social pain**						
NS vs. SKS	648.30	<.001	20.17	<.001	17.08	<.001
NS vs. NKS	44.83	<.001	2.59	.036	5.63	<.001
SKS vs. NKS	53.33	<.001	7.93	<.001	3.03	.016

Results of the contrasts between conditions following the overall ANOVAs across groups and separate t-tests within each of the experimental groups for the empathy for physical pain task and empathy for social pain task. F-CG, non-clinical control group; F-ASD, autism spectrum disorder group; PP, physical pain situations; NP, no-pain, neutral control stimuli; NS, neutral control situations; SKS, shared knowledge situations (see Methods for description); NKS, non-shared knowledge situations; EPP degrees of freedom: F_(1,16)_; ESP degrees of freedom: F_(1,15)_.

**Figure 2:**
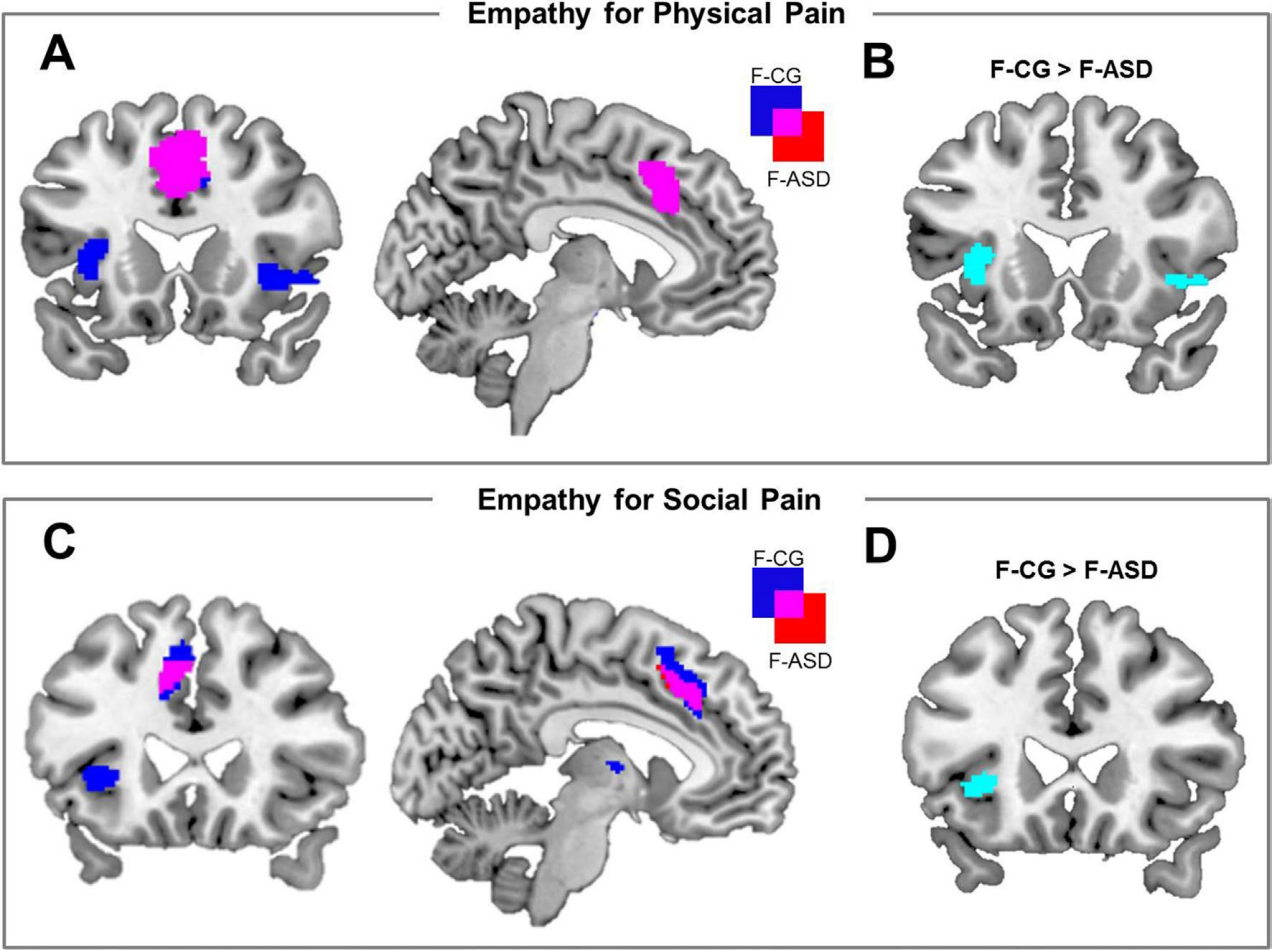
Neural activation associated with empathy for physical pain and empathy for social pain and activation differences between groups. **(A)** Brain activation for the main effect of physical pain empathy [physical pain (PP) − no-pain (NP)] for females with autism spectrum disorders (F-ASD) and non-clinical controls (F-CG). **(B)** Reduced activation of the bilateral anterior insula in response to PP vs. NP in the F-ASD group as compared to F-CG. **(C)** Neural activation for the main effect of social pain empathy {[shared knowledge situations (SKS) − neutral control situations (NS)] + (non-shared knowledge situations (NKS) − NS)} for F-ASD and F-CG. **(D)** Reduced activation of the left anterior insula in response to social pain empathy [(SKS − NS) + (NKS − NS)] in the F-ASD group as compared to F-CG; all statistics are FWE-corrected within ROIs as described in the Methods section; results are presented uncorrected (*p* < .05; *T* > 1.69 for EPP and *T* > 1.69 for ESP) for displaying purposes within ROIs.

On the neural systems level, participants of both groups showed increased activations of the ACC during PP compared to NP (F-CG at −2, 26, 46, *t_(32)_* = 6.44, *p* < .001, *k* = 587; F-ASD at −6, 18, 36, *t_(32)_* = 5.12, *p* = .002, *k* = 407; see **Figure 2A** and **Table 3**). The F-CG group additionally showed increased activations in the left AI (−36, 18, 0, *t_(32)_* = 5.91, *p* < .001, *k* = 156), right AI (42, 18, −4, *t_(32)_* = 4.59, *p* = .001, *k* = 110), and left somatosensory cortex (−58, −28, 36, *t_(32)_* = 3.77, *p* = .013, *k* = 42). Whole-brain analysis revealed an additional activation of the middle frontal gyrus in response to PP vs. NP of the F-CG group (see **Supplementary Table S1**). Comparing the F-ASD group to the F-CG group, the F-ASD showed significantly lower activation of the left AI (−34, 20, 4, *t_(32)_* = 3.28, *p* = .024, *k* = 16) and right AI (34, 24, 0, *t_(32)_* = 3.12, *p* = .033, *k* = 1) during PP vs. NP (see **Figure 2B**).

**Table 3:** Regions of interest (ROIs) analyses.

Brain region		Side	Cluster size	MNI coordinates			*T*	*p*	*p*FWE
				x	y	z			
**Empathy for physical pain**									
*PP > NP*									
F-CG									
	Anterior cingulate		587	−2	26	46	6.44	<.001	<.001
	Anterior insula	L	156	−36	18	0	5.91	<.001	<.001
	Anterior insula	R	110	42	18	−4	4.59	<.001	.001
	Somatosensory cortex	L	42	−58	−28	36	3.77	<.001	.013
									
F-ASD									
	Anterior cingulate		407	−6	18	36	5.12	<.001	.002
									
*F-CG (PP > NP) > F-ASD (PP > NP)*									
	Anterior insula	L	16	−34	20	4	3.28	<.001	.024
	Anterior insula	R	1	34	24	0	3.12	<.001	.033
									
**Empathy for social pain**									
*(SKS+NKS) > NS*									
F-CG									
	Anterior cingulate		222	−6	28	42	5.76	<.001	<.001
	Anterior insula	L	33	−28	24	0	5.49	<.001	<.001
	Thalamus		18	−2	−6	8	4.32	<.001	<.001
									
F-ASD									
	Anterior cingulate		25	−8	18	40	4.11	<.001	.005
									
*SKS > NKS*									
F-CG									
	Anterior insula	L	3	−28	24	2	2.83	<.001	.030
	Thalamus		23	−4	−6	6	3.76	<.001	.002
									
F-ASD									
	Anterior insula	L	2	−40	24	4	2.72	<.001	.039
	Thalamus		16	−6	−8	8	3.25	<.001	.006
									
*F-CG [(SKS+NKS) > NS] > F-ASD [(SKS+NKS) > NS]*									
	Anterior insula	L	22	−28	24	0	3.73	<.001	.003

All statistics for the contrasts reported in the table are thresholded at p < .05, family-wise-error corrected (FWE), within regions of interest (ROIs). PP, physical pain condition; NP, no pain condition; SKS, shared knowledge social pain situations; NKS, non-shared knowledge social pain situations; NS, neutral social control situations; F-CG, non-clinical control group; F-ASD, autism spectrum disorder group.

### Empathy for Social Pain

**Figure 3:**
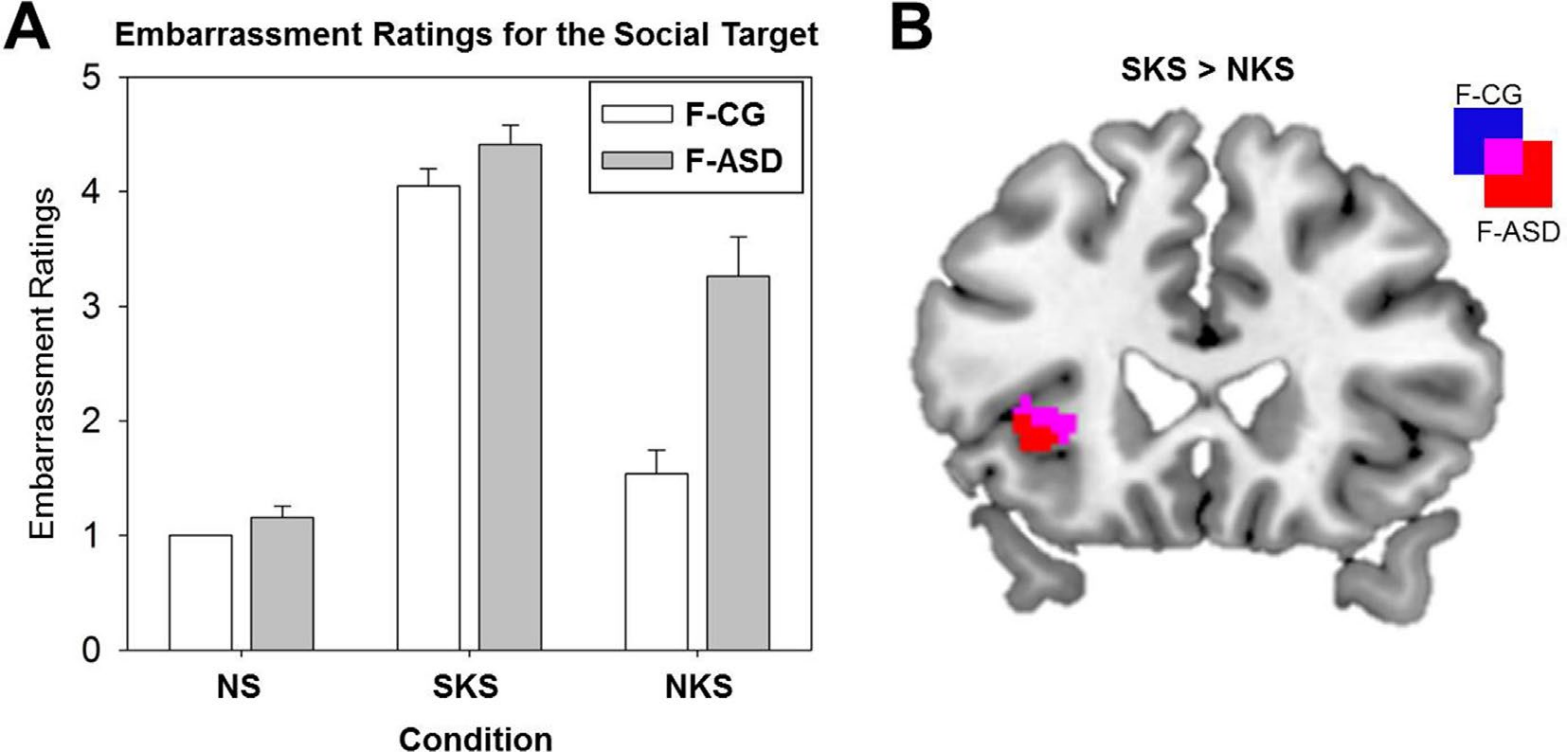
Ratings of the social targets’ experience of embarrassment and neural activation differences between shared and non-shared knowledge situations. **(A)** Mean subjective ratings of the social targets’ embarrassment (bars indicate standard errors) for the neutral control situations (NS), the shared knowledge situations (SKS), and the non-shared knowledge situations (NKS) for the control group (F-CG) and the female autism spectrum disorder group (F-ASD). **(B)** Increased activation of the left anterior insula during SKS in contrast to NKS in the F-CG and F-ASD group; all statistics are FWE-corrected within ROIs as described in the Methods section; all results are presented uncorrected (*p* < .05; *T* > 1.68) for displaying purposes within ROIs.

Behavioral results showed a significant main effect of Condition for the ratings of the social targets’ embarrassment (*F*_(2,30)_ = 128.36, *p* < .001). The social targets’ embarrassment was rated stronger during SKS and NKS compared to NS (see **Figure 3A** and **Table 2** for detailed comparisons). However, as expected, ratings of the social targets’ experience of embarrassment were decreased for NKS, during which the social target was unaware of the ongoing norm violation, and thus not experiencing embarrassment as compared to SKS. Separate *t*-tests for both groups showed that F-CG and F-ASD rated the social target’s embarrassment lower in NKS vs. SKS situations indicating an adjustment of their egocentric perspective towards the social target’s affective state. There was a main effect of Group (*F*_(1,15)_ = 21.32, *p* < .001) and a significant interaction of Group and Condition (*F*_(1,15)_ = 9.32, *p* = .001). This indicates that F-ASD rated the social target’s embarrassment higher than F-CG, particularly in the NKS compared to the SKS condition (*F*_(1,15)_ = 7.41, *p* = .016; F-ASD (SKS vs. NKS) vs. F-CG (SKS vs. NKS); significant for Bonferroni corrected *p*-level: *p* = .05/3 = .017) pointing towards a stronger egocentric bias in F-ASD as compared to F-CG.

On the neural level, embarrassment situations compared to NS were associated with increased activations within the ACC and AI network. Contrasting SKS and NKS vs. NS [(SKS − NS) + (NKS − NS)] resulted in increased activations of the left ACC in both groups (F-CG at −6, 28, 42, *t_(48)_* = 5.76, *p* < .001, *k* = 222; F-ASD at −8, 18, 40, *t_(48)_* = 4.11, *p* = .005, *k* = 25; see also **Table 3**). The left AI (−28, 24, 0, *t_(48)_* = 5.49, *p* < .001, *k* = 33) and thalamus (−2, −6, 8, *t_(48)_* = 4.32, *p* < .001, *k* = 18) showed increased activation in the F-CG group (see **Figure 2C**). For the same contrast, whole-brain analysis revealed additional activation of the left posterior medial frontal cortex in the F-CG group (see **Supplementary Table S1**). For both groups, the activation of the AI was decreased during NKS compared to SKS (F-CG at −28, 24, 2, *t_(48)_* = 2.83, *p* = .030, *k* = 3; F-ASD at −40, 24, 4, *t_(48)_* = 2.72, *p* = .039, *k* = 2; see **Figure 3B**), as well as activation of the thalamus (F-CG at −4, −6, 6, *t_(48)_* = 3.76, *p* = .002, *k* = 23; F-ASD at −6, −8, 8, *t_(48)_* = 3.25, *p* = .006, *k* = 16). Whole-brain analysis also revealed decreased activation of the ACC during NKS compared to SKS in the F-ASD group (see **Supplementary Table S1**).

When comparing the F-ASD group to the F-CG group, activation of the left AI was significantly lower when contrasting SKS and NKS vs. NS (−28, 24, 0, *t_(48)_* = 3.73, *p* = .003, *k* = 22; see **Figure 2D**). This effect was present for SKS vs. NS (−28, 24, 0, *t_(48)_* = 3.23, *p* = .012, *k* = 9) and also NKS vs. NS (−28, 24, 0, *t_(48)_* = 3.30, *p* = .010, *k* = 23), replicating previous findings of decreased responses during embarrassment on behalf of others in ASD. Unlike the behavioural data, fMRI data showed no significant difference between groups when contrasting SKS vs. NKS within the regions of interest or in the whole-brain analysis (even with more lenient threshold of *p* = .001, uncorrected).

## Discussion

The current study targeted the female variant of ASD: by using ecologically valid and emotionally complex social scenes, we aimed to characterize behavioral and neurobiological markers of empathy for physical and social pain in nine females with ASD compared to a carefully matched control group. To our knowledge, this is the first study investigating empathy for physical and social pain in this way in a group of female participants affected by ASD. A general lack of research on the female phenotype poses a challenge to the generalizability of the notion that alterations of processes in the domain of social cognition, such as emotion recognition, empathy, and theory of mind, could explain the observed peculiarities in social interactions in ASD. With the current study, we aimed to broaden our perspective on the ASD phenotype by testing deficits in empathic responding as well as egocentric biases in a female sample in correspondence to what has already been discussed for males with ASD. We found that females with ASD were able to correctly detect another person’s physical pain but were significantly less likely to accurately consider the protagonist’s perspective in case of social pain. In contrast to non-clinical control participants, they attributed relatively strong embarrassment to the social target even in a situation the person was unaware about the ongoing threat to his or her social integrity. In addition, fMRI results indicated an attenuated activation of areas of the empathy network, specifically the AI, in females with ASD, which persisted regardless of the complexity of the social situation. In both the empathy for physical pain and the more complex empathy for social pain condition, the AI did not show elevated activity in response to the depicted integrity threat.

The anterior insula cortex is typically associated with social-emotional processing, such as interoceptive processes (40–42), and empathizing with others conditions (43–46). In light of this popular notion of the AI’s functioning in social-affective processes, attenuated activity in the AI in females with ASD in response to another person’s physical and social pain might reflect deficits in sharing another’s perspective. Here, individuals with ASD might lack the intuitive access to another person’s mind through automatically sharing their affective states on the neural systems level. Thus, they might be less able to rely on their “gut feelings” in situations involving empathic abilities. Nevertheless, the females with ASD did actively try to “walk in the protagonists shoes” and were able to cognitively grasp and understand the other person’s situation (if they shared the same knowledge about the situation) as indicated by the ratings that were in a similar range as the non-clinical controls’. This notion is in line with previous studies suggesting that male patients with ASD might be able to make up for their lack of intuitive access to the shared representation of affect by adhering to learned social rules and conventions—however, often in an inflexible or stereotyped manner (13, 47).

This seemingly straightforward interpretation of altered AI responses in females with ASD, however, needs to be carefully considered, since the AI is well established in various other processes that are relevant for social behavior, most prominently salience (48). Reduced AI activation could refer to a qualitatively different level of social processes such as a reduced distribution of attention to social events in the environment in females with ASD. Reduced salience of social norm violations in the environment or threats to the physical integrity of another’s body could very well explain different behavioral responses in the absence of any alterations in shared representation of affect (49). Females with ASD, however, did notice the violation of social norms in the observed situation and rated the protagonist’s embarrassment just as high as the control participants—even higher in the non-shared knowledge condition—which points to the idea that the depicted norm violations are comparably salient to the females with ASD in this study. This is also supported by clinical observations and the importance of behavioral rules (47) and capabilities in understanding and realizing social norm transgressions in the high-functioning phenotype (50), particularly when these are very simple and do not rely on understanding another’s intentions (18). The activation differences in the AI as observed here might therefore rather unlikely reflect alterations in the ascription of saliency to the social scenarios.

In contrast to our expectations derived from previous findings in male subjects, in the current female sample, deficits in sharing another person’s state have surfaced even when participants were confronted with rather simple physical pain scenarios that did not require complex integrations of social context information with different social perspectives. Rather than indicating specific deficits in the female ASD phenotype, these more pronounced deficits in shared representation might be due to the younger age and thus earlier stage of maturation of the females in our sample compared to previous male samples (13). A previous study even pointed towards female superiority with respect to developmental aspects in younger individuals (non-clinical controls and individuals with ASD) when detecting faux pas in a theory of mind task (51). In this study, boys and girls aged 4–6 and 7–11 years were asked to detect a faux pas from a story. Besides a general effect of age (older children detected a faux pas better than younger children), girls outperformed boys in both age groups. The authors find faux pas detection performance to be at a lower level in children with high functioning autism compared to non-clinical controls. However, the group of female participants with autism was too small (*N* = 2) to statistically test for sex effects (51). Since the present study included only female participants with ASD precluding direct comparison to males, future research needs to include both male and female individuals with ASD and directly assess behavioral and neural aberrations associated with the disorder.

While females with ASD did not show any different behavior in the physical pain condition, complex social scenarios posed a greater challenge. Specifically, when the situations required to leave the own perspective and to accurately consider the social target’s perspective, females with ASD exhibited increased egocentric biases. Interestingly, both groups of participants showed enhanced activation of brain regions of the empathy network when viewing situations depicting a social target being unaware of the faux pas in contrast to neutral situations and females with ASD also showed the same increase in activation in the ACC when the knowledge on the norm-transgression and affective experience was shared compared to when it was not. This might reflect the vicarious embarrassment that participants experienced on behalf of the observed person as indicated by previous studies and might not be fundamentally different also in females with ASD (17, 23). However, the activation in the non-shared condition was particularly diminished in both groups in contrast to shared knowledge situations in which participants could feel the same affect as the social target. This indicates that the representation of non-shared, vicariously experienced embarrassment is less prominent when participants are explicitly asked to focus on the other’s mental states rather than their own affect on behalf of the other person. In this context, activations of the anterior insula might in part also reflect one’s own experience of distress when observing another person in an uncomfortable social situation (52). Such vicarious and subjectively imbued neural representations have been found in previous studies confronting individuals with a person that was “not like them” (27), supporting the view that perspective taking requires the effortful regulation of one’s egocentrically anchored experiences when another person feels different than oneself (26). While subjectively biased representations of another person’s state are thought be a typical phenomenon (30, 53), in the current study, individuals without ASD were fully able to cognitively understand the social target’s situation and make a clear distinction between the self and the other. Even though on the neural systems level participants without ASD represented the social pain on behalf of another to a stronger degree than females with ASD, they were also able to withdraw from this experience and to distinguish their own from the social target’s feeling state. Thus, non-clinical control participants did not confuse self- and other-related emotional responses. In contrast, when confronted with such complex social scenarios, it seemed to be difficult for subjects with ASD to take a step back from their own judgment of a situation and create a valid evaluation of someone else’s internal affective state. This finding is in line with clinical observations describing individuals with ASD as constantly observing themselves, directing their attention towards the own person, which results in an extreme form of egocentrism (29, 30). Naturally, such deficits in perspective taking in a social context may make it harder for individuals with ASD to react in a socially acceptable or culturally expected manner.

However, females with high functioning ASD did show activation of the same empathy networks as typically developed individuals, indicating that their ability to share another’s affect is not fundamentally compromised (54, 55). Behavioral deficits in understanding another person’s affective state only surfaced in more complex social settings that require disentangling different social perspectives. Thus, training of cognitive strategies, such as consultation of learned and memorized social relations, with an emphasis on perspective taking skills, might enable individuals with ASD to compensate some of these disadvantages and help them to get along in a social environment, even though they might not be able to intuitively feel it or “naturally” fit in.

### Limitations

In the present study, we did not assess alexithymia, i.e., the inability to describe one’s own affective states. In recent years, it has been observed that many individuals with ASD also suffer from alexithymia, leading to the assumption that emotional problems in ASD may rather be a symptom of (co-occurring) alexithymia than a core feature of the disorder itself (56). For example, it was suggested that processing of physical pain, sensitivity to subjective experiences of physical pain, or the report thereof may be affected in ASD (57). According to this reasoning, the attenuated AI activation in ASD in the present study may emerge because of deficits in processing pain per se, but not attributed to deficits in perspective-taking (58). This is in line with other studies in ASD demonstrating that insular activity varied over time during pain stimulation, with unobtrusive early but diminished late responses to physical pain (57). In sum, although there are studies showing no contribution of alexithymia to impairment in emotion processing (59), others highlight a mediating role of alexithymia on empathic processes in ASD (60). These conflicting findings call for more careful investigations of specific subgroups of individuals with co-occurring ASD and alexithymia. Regarding the interpretation of our results, the lack of information on alexithymia in the present sample thus needs to be considered.

Future research should take into account potential influences of age, sex/gender, and hormone status, e.g., female cycle, intake of oral contraceptives, oxytocin and vasopressin levels, and stress hormone levels, as studies show connections between these factors and social behavior, including empathic abilities, in humans (61, 62). Within this line, particularly the large age range within this study (12–24 years) needs to be discussed, as particularly within this period of development, substantial changes in biology and cognition may take place. Interpretation of the present results is further limited by the small sample size.

## Conclusion

Our findings point towards a tendency of reduced shared representations of affect in empathic situations in females with high functioning ASD. While females with ASD do experience shared affect, they tend to differentiate less between the own and another person’s perspective. The overreliance on their own perspective confirms the notion that deficits in understanding another’s feelings might only surface in more complex social settings that require disentangling the own from another person’s view of the situation. These findings fit very well into previous literature on the male ASD phenotype and suggest that the peculiarities in the domain of social cognition also generalize to the female phenotype (13). The here presented evidence yet relies on a small sample of high functioning females with ASD and should thus be treated with caution. Considering the strong asymmetry of males and females in high functioning ASD, however, we strongly believe that these efforts are inevitable and a valuable addition to the literature to obtain a more comprehensive understanding of the generalizability of altered social cognition in the heterogeneous ASD phenotype.

## Ethics Statement

This study was carried out in accordance with the recommendations of the ethics committee of the Medical Department of the University of Marburg with written informed consent from all subjects. All subjects gave written informed consent in accordance with the Declaration of Helsinki. The protocol was approved by the ethics committee of the Medical Department of the University of Marburg (Az 197/12).

## Author Contributions

LP, IK-B, FMP, SK, and LM-P acquired the data. SS, LP, and LM-P analyzed the data. SS, LP, IK-B, A-KW, FMP, SK, and LM-P prepared, reviewed, and edited the manuscript.

## Funding

Research leading to this publication was funded by the German Research Foundation (KR3803/2-1, KR3803/7-1, MU 4373/1-1), the Research Foundation of the Philipps-University Marburg, the von Behring-Roentgen-Stiftung (KR 60-0023).

## Conflict of Interest Statement

The authors declare that the research was conducted in the absence of any commercial or financial relationships that could be construed as a potential conflict of interest.

## Footnotes

1 Negative affective experiences in response to socially unpleasant situations like social exclusion or embarrassment are considered a form of social pain (17, 19–22). The term of social pain has been established to emphasize the conceptual overlap of the distress and affective arousal of social rejection or embarrassment with physical pain. While physical pain is thought to signal a threat for the bodily integrity of the individual, the affective experiences in the domain of social pain are thought to serve a similar function, signaling threats to the social integrity of individuals that are being excluded from or judged negatively by their social group (17, 20).
