# Supplementary material for: Empathy in Females With Autism Spectrum Disorder
Source: Front Psychiatry. 2019 Jun 18;10:428. doi: 10.3389/fpsyt.2019.00428 (PMC6591689; doi:10.3389/fpsyt.2019.00428)
Supplement: Supplementary file 1 [file Table_1.docx]

**Supplementary Information**

**Supplementary Tables**

| *Table S1*. Additional whole-brain analyses | | | |  |  |  |  |  |  |
| --- | --- | --- | --- | --- | --- | --- | --- | --- | --- |
| Brain Region | | Side | Cluster Size | MNI Coordinates | | | *T* | *p* | *p*FWE |
|  |  |  |  | x | y | z |  |  |  |
| **EPP** | |  |  |  |  |  |  |  |  |
| F-CG: PP > NP | |  |  |  |  |  |  |  |  |
|  | Middle Frontal Gyrus | L | 12 | -40 | 48 | 14 | 6.62 | < .001 | .005 |
|  | Superior Medial Gyrus/ | L/R | 95 | -2 | 26 | 46 | 6.44 | < .001 | .008 |
|  | Anterior Cingulate |  |  | -42 | 14 | 52 | 6.10 | < .001 | .004 |
|  |  |  |  |  |  |  |  |  |  |
| **ESP** | | | |  |  |  |  |  |  |
| F-CG: (SKS+NKS) > NS | |  |  |  |  |  |  |  |  |
|  | Posterior Medial Frontal | L | 50 | -10 | 20 | 62 | 6.49 | < .001 | .001 |
|  | Superior Medial Gyrus/ | L | 28 | -6 | 28 | 42 | 5.76 | < .001 | .010 |
|  | Anterior Cingulate |  |  |  |  |  |  |  |  |
|  |  |  |  |  |  |  |  |  |  |
| F-ASD: SKS > NKS | |  |  |  |  |  |  |  |  |
|  | Superior Medial Gyrus/ | R/L | 40 | 0 | 58 | 16 | 5.67 | < .001 | .014 |
|  | Anterior Cingulate |  |  |  |  |  |  |  |  |
|  |  |  |  |  |  |  |  |  |  |
| *Note.* All statistics for the contrasts reported in the table are thresholded at p < .05, family-wise-error corrected (FWE), for the whole-brain. EPP = empathy for physical pain. PP = physical pain condition. NP = no pain condition. ESP = empathy for social pain. SKS = shared knowledge social pain situations. NKS = non-shared knowledge social pain situations. NS = neutral social control situations. F-CG = non-clinical control group. F-ASD = autism spectrum disorder group. | | | | | | | | | |
|  |  |  |  |  |  |  |  |  |  |
|  |  |  |  |  |  |  |  |  |  |
|  |  |  |  |  |  |  |  |  |  |
